# Metabolic adaptations during extreme anoxia in the turtle heart and their implications for ischemia-reperfusion injury

**DOI:** 10.1038/s41598-019-39836-5

**Published:** 2019-02-26

**Authors:** Amanda Bundgaard, Andrew M. James, Anja V. Gruszczyk, Jack Martin, Michael P. Murphy, Angela Fago

**Affiliations:** 1 Department of Bioscience, Aarhus University, Aarhus, Denmark; 2 MRC Mitochondrial Biology Unit, University of Cambridge, Cambridge, UK

## Abstract

ATP depletion and succinate accumulation during ischemia lead to oxidative damage to mammalian organs upon reperfusion. In contrast, freshwater turtles survive weeks of anoxia at low temperatures without suffering from oxidative damage upon reoxygenation, but the mechanisms are unclear. To determine how turtles survive prolonged anoxia, we measured ~80 metabolites in hearts from cold-acclimated (5 °C) turtles exposed to 9 days anoxia and compared the results with those for normoxic turtles (25 °C) and mouse hearts exposed to 30 min of ischemia. In turtles, ATP and ADP decreased to new steady-state levels during fasting and cold-acclimation and further with anoxia, but disappeared within 30 min of ischemia in mouse hearts. High NADH/NAD^+^ ratios were associated with succinate accumulation in both anoxic turtles and ischemic mouse hearts. However, succinate concentrations and succinate/fumarate ratios were lower in turtle than in mouse heart, limiting the driving force for production of reactive oxygen species (ROS) upon reoxygenation in turtles. Furthermore, we show production of ROS from succinate is prevented by re-synthesis of ATP from ADP. Thus, maintenance of an ATP/ADP pool and low succinate accumulation likely protects turtle hearts from anoxia/reoxygenation injury and suggests metabolic interventions as a therapeutic approach to limit ischemia/reperfusion injury in mammals.

## Introduction

Mammalian hearts tolerate only short periods without blood oxygen supply (ischemia) and suffer extensive oxidative damage when reoxygenated at reperfusion (I/R injury). In contrast to mammals, some turtles hibernate during winter in the complete absence of oxygen^1,2^ and species like *Trachemys scripta* are highly anoxia-tolerant and can survive several weeks without oxygen at low temperatures^3,4^, relying completely on glycogen reserves for energy production^1,3–6^. Lactate, the end product of anaerobic energy metabolism, accumulates in cells, allowing regeneration of cytosolic NAD^+^ to support ATP production^7^. It is then exported to the plasma, where it is buffered by calcium carbonate released from the shell^8^. While most other organs almost completely shut down metabolism during anoxia, the heart keeps beating (albeit at low rate) to circulate nutrients, lactate and other waste products^7^. Thus, freshwater turtles provide valuable models to study long-term remodelling of energy metabolism when a heart is completely anoxic for an extended period of time. This enables comparison of naturally evolved mechanisms of physiological tolerance to anoxia and reoxygenation that may be of relevance to the pathology of ischemia and reperfusion.

During ischemia, succinate, a citric acid cycle (CAC) intermediate, accumulates in mammalian hearts as it cannot be oxidised by the mitochondrial respiratory chain^9^. Upon reoxygenation, succinate oxidation fuels an overproduction of mitochondrial reactive oxygen species (ROS), causing extensive cardiac damage^9^. This arises when electrons from succinate oxidation by complex II are forced backwards through complex I by a high mitochondrial proton-motive force. This process is known as reverse electron transfer (RET) and is favoured when ATP generation by the ATP synthase is low^10,11^. At the flavin site of complex I electrons can react with molecular oxygen, forming superoxide, which is converted further to H_2_O_2_. Succinate is thus a major cause of oxidative damage in the mammalian heart after I/R. Although one report found accumulation of succinate in the heart of a turtle species during anoxia^12^, the heart of *Trachemys* and of other turtle species does not suffer from oxidative damage when reoxygenated^13,14^, and it is unclear how they avoid this damage.

To investigate how freshwater turtles regulate their metabolism to survive prolonged anoxia and subsequent reperfusion, we used liquid chromatography-mass spectrometry (LC-MS) to compare metabolites present in heart ventricles from fasted turtles acclimated to 5 °C and exposed to normoxia or anoxia with those of mouse hearts before and after acute ischemia. To distinguish between metabolic changes that happen during acclimation to low temperatures, which is associated with fasting, and those that occur during anoxia, we used fed, normoxic turtles kept at 25 °C for comparison. Here we show that part of its remarkable metabolic tolerance to extreme oxygen deprivations likely occurs because the anoxic turtle heart accumulates less succinate and maintains higher adenylate pools. This allows turtle mitochondria to rapidly resume oxidative phosphorylation upon reperfusion, preventing the increase in mitochondrial membrane potential and consequent ROS production at reoxygenation.

## Results

### Fatty acids and amino acids

**Figure 1:**
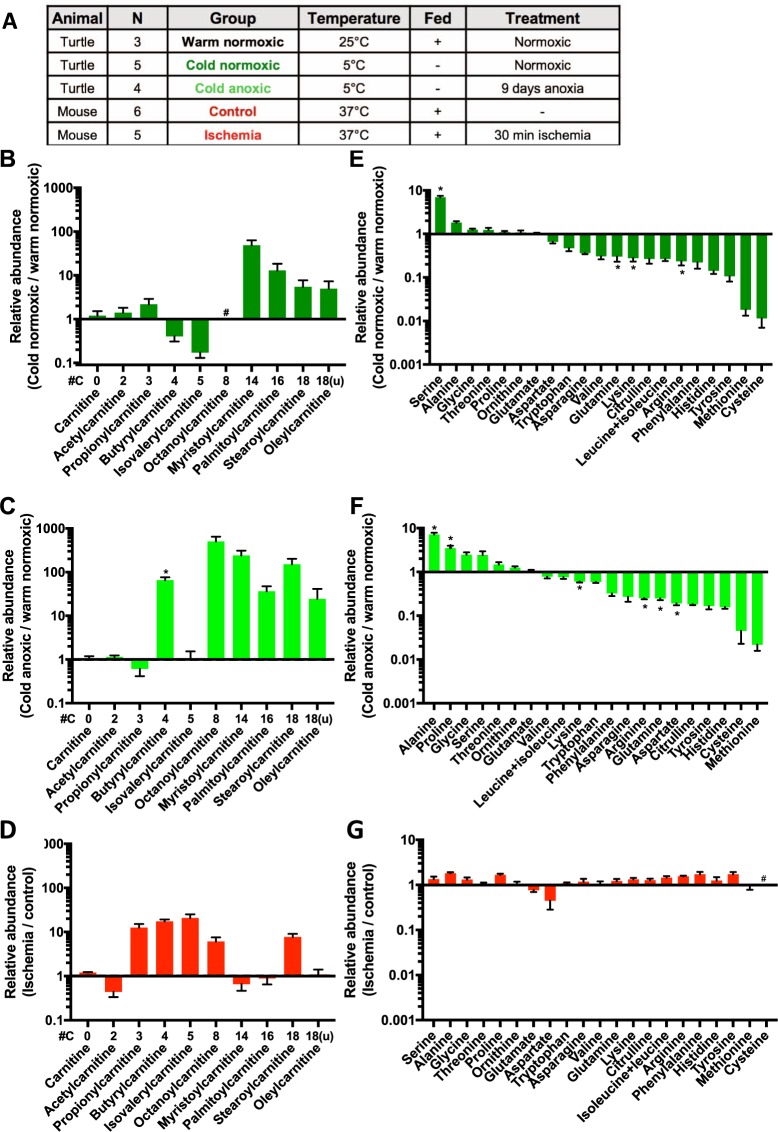
Experimental design and changes in fatty acid and amino acid metabolism in heart ventricles upon cold acclimation and anoxia in turtles and ischemia in mouse. (**A**) Overview of experimental groups. Relative changes in (**B**–**D**) fatty acid carnitines and (**E**–**G**) free amino acids. ^#^Not detected in both compared groups. *p < 0.05 compared to control, as indicated.

Turtles were fasted and acclimated to low temperature (5 °C) and exposed to anoxia or normoxia for 9 days^13^, or kept at 25 °C and fed *ad libitum*. An overview of the turtle and mouse treatments is given in Fig. 1A. The full LC-MS datasets and analyses are given in Supplementary Tables 1 and 2, respectively. LC-MS metabolomic analyses of heart ventricles showed that cold-acclimation led to an accumulation of long-chain fatty acyl (C14-C18) carnitines in normoxic turtles (Fig. 1B), and long-chain fatty acyl carnitines increased further when turtles were exposed to anoxia (Fig. 1C). This metabolic shift towards fatty acid utilisation during fasting and cold agrees with previous results in turtles^15^ and other reptiles^16,17^. The large increase in butyrylcarnitine during anoxia with no change in acetylcarnitine (Fig. 1C) suggests that the enzymes upstream of the acetyl-CoA formation step of fatty acid β-oxidation are inactive during anoxia. A decrease of acetylcarnitine and an accumulation of other short or medium-chain (C4-C8) fatty acylcarnitines (butyryl- and octanoylcarnitine) was also seen in the ischemic mouse heart (Fig. 1D), as β-oxidation of fatty acids cannot proceed when oxygen is lacking. These results are consistent with the expected shift to carbohydrate fuel utilization during anoxia, when fatty acid oxidation is impeded^18^. Propionyl- and isovalerylcarnitine derive from distinct amino acid degradation pathways and thus have different susceptibilities to anoxia.

There was a general decrease of most free amino acids in turtle heart with cold-acclimation and fasting, with the exception of the 7-fold increase of serine (Fig. 1E). Upon exposure to anoxia, levels of alanine, proline, valine, lysine and leucine/isoleucine increased while serine and aspartate levels decreased (Fig. 1F). As most amino acid levels did not change significantly over 30 min of ischemia in mouse heart (Fig. 1G), these long-term changes in free amino acids probably reflect increased protein turnover in turtles in response to starvation and anoxia.

### Adenine nucleotides

**Figure 2:**
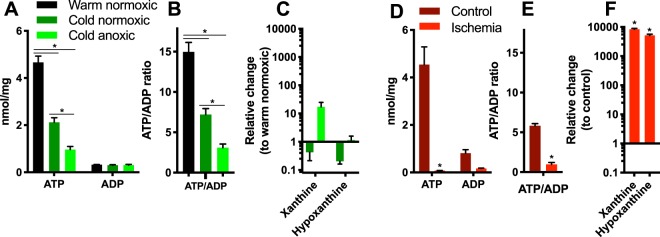
ATP and ADP levels and depletion products xanthine and hypoxanthine in the turtle heart upon cold acclimation and anoxia and in the ischemic mouse heart. Turtle heart (**A**) absolute ATP and ADP concentration, (**B**) ATP/ADP ratio and (**C**) changes in xanthine and hypoxanthine relative to warm normoxic turtles. Mouse heart (**D**) absolute ATP and ADP concentration, (**E**) ATP/ADP ratio and (**F**) changes in xanthine and hypoxanthine relative to control mice. Statistical details as in Fig. 1.

ATP levels (Fig. 2A) and the ATP/ADP ratio (Fig. 2B) decreased significantly in the anoxic turtle heart, but the adenylate nucleotide pool was maintained even after 9 days of anoxia and the products of adenylate nucleotide degradation, xanthine and hypoxanthine, remained low (Fig. 2C), consistent with a new steady-state of adenylate phosphorylation. In contrast, ATP was entirely consumed and the purine nucleotide pools depleted within 30 min (Fig. 2D) in the ischemic mouse heart. Furthermore, the adenylate degradation products xanthine and hypoxanthine accumulated ~10000 fold during ischemia compared to controls (Fig. 2F) in the mouse heart.

### Glycolysis

**Figure 3:**
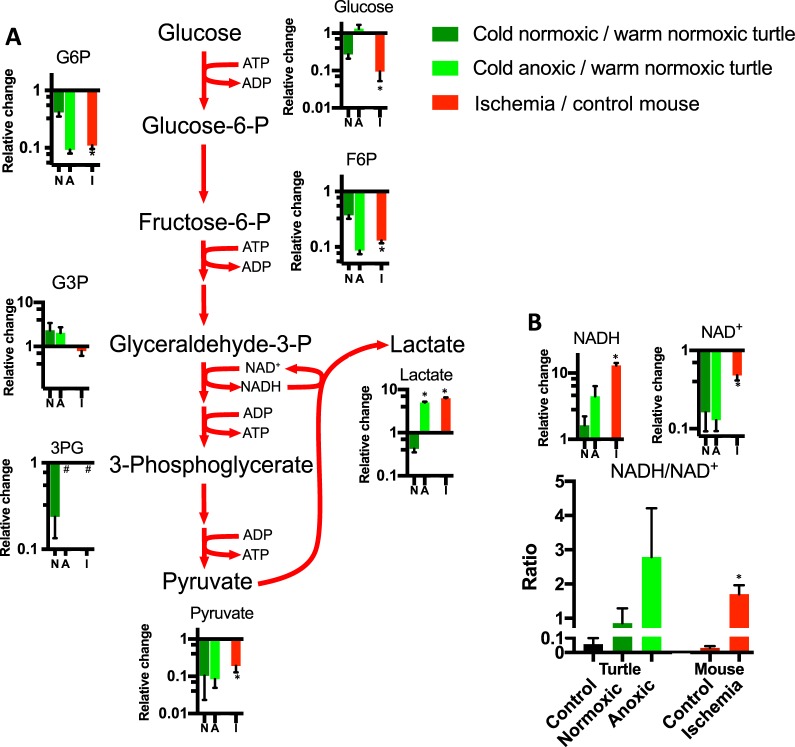
Relative changes of (**A**) glycolytic intermediates and (**B**) NADH and NAD^+^ in the turtle heart upon cold acclimation and anoxia and in the mouse heart upon ischemia, as indicated. Data are presented relative to warm normoxic turtles and control mouse hearts, respectively. An increase in metabolite levels results in a relative change >1, whereas a decrease results in a relative change <1. Statistical details as in Fig. 1.

Fasting and cold-acclimation of turtles led to decreases in most glycolytic intermediates in the heart (Fig. 3A), including glucose and pyruvate, reflecting decreased glycolytic activity. The lowered levels of lactate during fasting (Fig. 3A) suggests that it acts as a substrate as long as oxygen is present^19,20^.

In the absence of oxygen, the ischemic mouse heart must rely on glycolytic breakdown of glucose derived from its own limited glycogen stores for ATP production. The ~10-fold decrease in glucose compared to control suggests that these glycogen stores are depleted rapidly (Fig. 3A). In contrast, continuing blood supply from the circulation would allow the turtle heart access to glucose from glycogenolysis of liver glycogen stores, maintaining glucose levels during anoxia (Fig. 3A). Despite these differences, the glycolytic intermediates glucose-6-phosphate, fructose-6-phosphate, 3-phosphoglycerate and pyruvate decreased to a similar extent in both anoxic turtle hearts and ischemic mouse hearts (Fig. 3A). Glyceraldehyde-3-phosphate was an exception as its levels increased slightly in the anoxic turtle heart and remained constant in the ischemic mouse heart (Fig. 3A). Widespread loss of downstream 3-phosphoglycerate (Fig. 3A) and phosphoenolpyruvate (Supplementary Table 2) suggests that glyceraldehyde 3-phosphate dehydrogenase and/or phosphoglycerate kinase are inhibited, possibly by low levels of adenine nucleotides (Fig. 2A) or increased NADH (Fig. 3B).

Regeneration of NAD^+^ by lactate dehydrogenase leads to a similar ~5-fold lactate accumulation in both anoxic turtle and ischemic mouse hearts (Fig. 3A). However, in contrast to the ischemic mouse heart, the lactate produced by the anoxic turtle heart can be exported to the circulatory system to be buffered by calcium carbonate in the plasma and shell^21^. Even so, the ratio of ion counts from NADH relatively to NAD^+^ increased in the turtle heart from 0.05 ± 0.03 in the warm normoxic turtle to 0.86 ± 0.43 after fasting and cold-acclimation and further to 2.78 ± 1.42 with anoxia. Surprisingly, this increase was more pronounced than in the mouse heart, where the NADH/NAD^+^ ion count ratio changed from 0.04 ± 0.01 in controls to 1.70 ± 0.26 during ischemia (Fig. 3B). These changes resulted from the accumulation of NADH rather than the depletion of NAD^+^ (Supplementary Table 1) most likely reflecting an increase in cytosolic NADH, given the large cytosolic volume of the cell.

Together, the results so far suggest that turtles are able to match low glycolytic ATP generation with low ATP requirements during anoxia and that this leads to lower steady-state ATP and ADP concentrations. In contrast, mouse hearts cannot meet their ATP requirements with ATP generation during ischemia, which leads to nearly complete adenine nucleotide degradation (Fig. 2D–F) and cessation of glycolysis.

### Citric acid cycle

**Figure 4:**
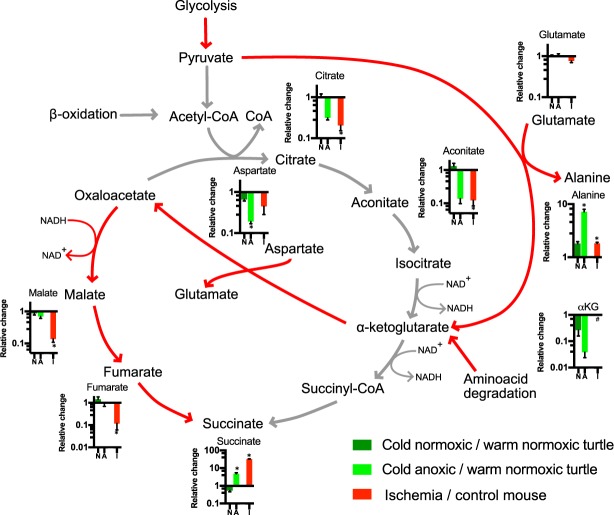
Relative changes of citric acid cycle intermediates in the turtle heart upon cold acclimation and anoxia and in the mouse heart upon ischemia, as indicated. Data are presented relative to warm normoxic turtles and control mouse hearts, respectively. Red lines: primary pathways in anoxia or ischemia; grey lines: downregulated pathways in anoxia or ischemia. An increase in metabolite levels results in a relative change >1, whereas a decrease results in a relative change <1. Statistical details as in Fig. 1.

In the absence of oxygen, the high NADH/NAD^+^ ratio (Fig. 3B) could cause remodelling of the CAC (Fig. 4). The levels of citrate and aconitate, intermediates in the conversion of oxaloacetate and acetyl-CoA to isocitrate, decreased markedly in the ischemic mouse heart and anoxic turtle heart (Fig. 4), consistent with inactivation of pyruvate dehydrogenase during oxygen deprivation. That alanine accumulated in the anoxic turtle heart (Fig. 4) suggests that pyruvate may bypass pyruvate dehydrogenase through alanine aminotransferase in the cytosol, producing alanine (which accumulates) and α−ketoglutarate (αKG) from glutamate (Fig. 4). In this scenario, αKG and aspartate can be used by aspartate amino transferase to generate oxaloacetate and regenerate glutamate, consistent with unchanged glutamate levels and depletion of aspartate during anoxia or ischemia (Fig. 4). In this model, oxygen deprivation and the high NADH/NAD^+^ ratio in the cytosol (Fig. 3B) favour generation of malate from oxaloacetate which can then be imported to the mitochondria thereby decreasing cytosolic NADH levels. Malate import leads to accumulation of succinate through fumarase and succinate dehydrogenase both working in reverse (Fig. 4). Malate and fumarate levels were maintained in the anoxic turtle heart, but were depleted in the ischemic mouse heart (Fig. 4).

### Succinate and fumarate

**Figure 5:**
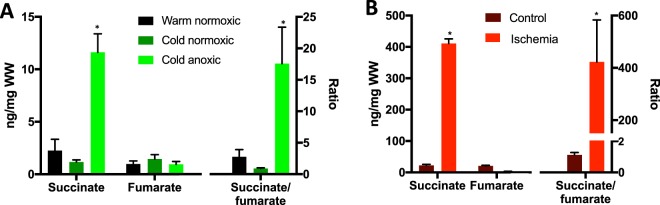
Absolute succinate and fumarate levels and succinate/fumarate ratios in (**A**) turtle hearts upon cold acclimation and anoxia and (**B**) mouse hearts upon ischemia. Note the different axes. Statistical details as in Fig. 1.

Succinate is known to drive ROS generation and I/R injury in mammals via RET^9,11^. While succinate increased ~20- and ~10-fold in ischemic mouse hearts and anoxic turtle hearts, respectively (Fig. 5A), the absolute succinate concentration in the heart of anoxic turtles was much lower (Fig. 5B). Moreover, the ratio of succinate to fumarate, providing the driving force for RET and ROS production at reoxygenation, was ~400 in the ischemic mouse heart and only ~15 in the turtle heart (Fig. 5A,B). Taken together, these data suggest a lower and less sustained driving force for RET-dependent ROS production from succinate during reoxygenation in the anoxic turtle heart compared to the ischemic mouse heart.

### ROS generation

**Figure 6:**
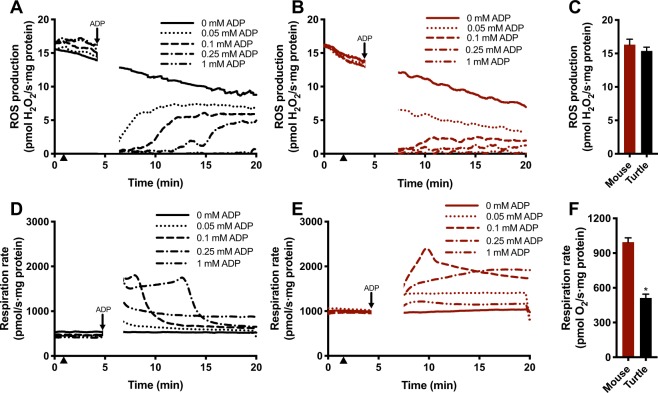
Effect of ADP on ROS production. Representative traces showing. (**A–C**) ROS production and (**D**–**E**) respiration rate in isolated turtle (shown in black) and mouse (shown in burgundy) heart mitochondria incubated with 10 mM succinate for 5 min inducing ROS production by RET. Addition of ADP reduced ROS production in a concentration dependent manner. When all ADP was converted to ATP, ROS production was resumed concomitant with a decrease in respiration rate. (**C**) Maximal ROS production by RET and (**F**) respiration rate at [O_2_] 250 µM (marked by arrow head) in isolated turtle and mouse heart mitochondria (N = 5 each). Statistical details as in Fig. 1.

ROS generation via RET requires an elevated mitochondrial membrane potential and mitochondrial ATP generation consumes this potential. The near complete loss of adenine nucleotides from ischemic mouse heart is likely to make ATP synthesis upon reperfusion initially difficult. In contrast, maintenance of adenine nucleotides in anoxic turtle hearts could allow the rapid resumption of mitochondrial ATP synthesis upon reoxygenation, thereby limiting RET. To test whether differences in adenine nucleotides between turtle and mouse could explain why turtles are resistant to oxidative injury we measured H_2_O_2_ production by isolated mitochondria in the presence and absence of ADP. When tested under identical conditions, i.e. with 10 mM succinate as substrate and in the absence of ADP, isolated heart mitochondria from warm normoxic turtles and from mice produce H_2_O_2_ at similar rates via RET (Fig. 6A–C), indicating they have a similar capacity for ROS production, despite different respiration rates (Fig. 6F). However, addition of ADP (0.05–0.25 mM) initially reduced H_2_O_2_ production (Fig. 6A,B) by dissipating the proton-motive force through ATP synthesis. H_2_O_2_ production was gradually restored over time (Fig. 6A,B) along with the concomitant decrease in respiration rate (Fig. 6D,E) as ADP was converted to ATP. This illustrates that succinate-dependent H_2_O_2_ production upon reoxygenation is strongly depressed by ADP availability.

Taken together, these data indicate a lower driving force for RET-dependent ROS production from succinate during reoxygenation in the anoxic turtle heart compared to the ischemic mouse heart.

## Discussion

This study provides new insights to the metabolic adjustments taking place when a vertebrate heart maintains its function, despite being deprived of oxygen for days, as occurs in the cold-acclimated anoxic turtles used in this study. Such extreme conditions are unattainable for the mammalian heart, which cannot tolerate even brief periods of ischemia. Here, we found that the ability of the turtle heart to survive extreme anoxia and subsequent reoxygenation originates from two major metabolic adaptations: 1) the ability to balance ATP synthesis and consumption during anoxia, thus avoiding degradation of ADP to AMP, xanthine and hypoxanthine; and 2) a limited accumulation of succinate and a lower succinate to fumarate ratio. Through these processes, the duration and severity of ROS production upon reoxygenation are limited.

ATP levels in the heart of warm normoxic turtles and mice are roughly similar (Fig. 2A,D), but ATP/ADP ratios are much higher in turtles than in mice (Fig. 2B,D). Although ATP and ATP/ADP ratios decrease in the turtle heart during acclimation to low temperature and further with exposure to anoxia (Fig. 2A,B), ADP levels are maintained (Fig. 2A). This suggests a new steady state for ATP and ADP is reached and that this is sufficient to support the energetic requirements of the heart during cold-acclimation as well as during anoxia. These results are in agreement with previous findings of high ATP/ADP ratios and unchanged ADP levels in anoxic turtle hearts^22–24^ and in another model of extreme anoxia tolerance, the crucian carp^25^. In addition, there is limited accumulation of the ATP degradation products xanthine and hypoxanthine during anoxia (Fig. 2C), which may reflect in part the export of purine degradation products to the circulation. Specifically, adenosine is known to function as an extracellular signalling molecule in turtles^5^. In contrast to anoxic turtle hearts, in the ischemic mouse heart most ATP and ADP are lost (Fig. 2D,E) and converted into xanthine and hypoxanthine (Fig. 2F). This difference reflects the remarkable ability of turtles to reduce energy demand to match the low energy production during long-term anoxia^5,6,26^. This metabolic depression, driven in part by the low body temperature, depends on active suppression of energy demand^5,6,26–28^.

How ATP production is downregulated in anoxia tolerant turtles and other anoxia tolerant ectotherms is poorly understood^5,22,29–32^. In the absence of oxygen, glycolysis is the only source of ATP production and glycolysis requires ADP to proceed. Thus, maintenance of ATP and ADP in the anoxic turtle heart (Fig. 2A-C) ensures substrates for phosphorylation are present, whereas in the ischemic mouse heart loss of adenine nucleotides (Fig. 2D–F) would contribute to glycolytic inhibition. In both ischemic mouse hearts and anoxic turtles, the levels of glycolytic intermediates decrease (Fig. 3A), while lactate accumulates (Fig. 3A) and the NADH/NAD^+^ ratio increases (Fig. 3B), indicating partial inhibition of glycolysis. Except for lactate, most of these changes in glycolysis occur already during acclimation to low temperatures in turtles, and are further enhanced during anoxia. Specifically, the increase in G3P and the concomitant decrease of downstream glycolytic intermediates (Fig. 3A) suggests that glyceraldehyde 3-phosphate dehydrogenase activity is inhibited (Fig. 3A), likely by a combination of increased NADH and lactate as is the case for the ischemic rat heart^33–35^. It is difficult to establish from our data whether phosphofructokinase, an important control enzyme in the glycolytic pathway, is allosterically inhibited *in vivo* by the decrease in pH^24^ or activated by the decrease in ATP (Fig. 2A) and citrate (Fig. 4). A previous study reported an overall increase in phosphofructokinase activity in the anoxic turtle heart^32^ but given the substantial drop in the availability of the substrate fructose 6-phosphate (Fig. 3A), it appears likely that its catalytic rate *in vivo* falls during anoxia. Further studies are required to fully understand how glycolytic rate is regulated during anoxia in the turtle heart.

The high NADH/NAD^+^ ratio during anoxia and ischemia (Fig. 3B) converts mitochondria to an NADH sink where fumarate instead is used as the final electron acceptor to generate succinate (Fig. 4). When oxygen is absent, pyruvate can either be converted into lactate or enter the CAC through reaction with glutamate catalysed by alanine aminotransferase producing alanine and αKG (Fig. 4), as found in hearts from diving animals^12,36,37^ and ischemic mammalian hearts^9,38^. This effectively bypasses the step catalysed by pyruvate dehydrogenase, whose activity is normally depressed by a specific kinase expressed during hypoxia^39^. As a consequence, the initial steps of the CAC catalysed by citrate synthase, aconitase and isocitrate dehydrogenase are also bypassed (Fig. 4), thus avoiding generation of NADH by isocitrate dehydrogenase (Fig. 4).

Our data indicates that the high NADH/NAD^+^ ratio (Fig. 3B) likely drives conversion of αKG to oxaloacetate and further to malate in the cytosol producing NAD^+^ in the process (Fig. 4). Mitochondrial malate import leads to accumulation of succinate through fumarase and succinate dehydrogenase working in reverse in both anoxic turtle and ischemic mouse hearts (Fig. 4), consistent with previous findings of succinate accumulation in turtles^12^ and mice^9^. Succinate can efflux from mitochondria in exchange for malate and the CAC thus becomes a sink for cytosolic NADH. The formation of succinyl-CoA from αKG may still contribute to succinate production during anoxia and ischemia, but this is likely inhibited by the high NADH/NAD^+^ ratio.

There are some notable differences between anoxic turtle and ischemic mouse hearts. Malate and fumarate levels decrease in the ischemic mouse heart (Figs 4 and 5B), whereas they are unchanged in the anoxic turtle heart (Fig. 4). Furthermore, succinate accumulation is less dramatic in the anoxic turtle than in the ischemic mouse heart (Figs 4 and 5). Hence, it is possible that succinate is removed from the cells and exported to blood perfusing the heart^40^. This is consistent with the appearance of succinate in the blood of *Chrysemys* turtles after 28 days of anoxia at 5 °C^12^. Exchange of succinate and malate via the dicarboxylate carrier would allow this process to continue if a sink for succinate outside the heart exists. Another factor that contributes to the limited succinate accumulation as well as limiting ROS production in turtles is the low mitochondrial content in ecto- versus endotherms^41–43^.

Because both the absolute and fold-accumulation of succinate as well as the increase in the succinate/fumarate ratio is much less dramatic in the anoxic turtle heart compared to the ischemic mouse heart (Fig. 5), the thermodynamic potential for ROS production via succinate upon reoxygenation is much lower in the turtle heart than in the mouse. Importantly, the maintenance of constant ADP levels (Fig. 2A) would allow the inner mitochondrial membrane potential to be dissipated through ATP production by ATP synthase upon reoxygenation, preventing extensive ROS production from succinate through RET (Fig. 6). Thus, the ability of the turtle heart to maintain energy balance in the absence of oxygen limits the potential for ROS production and oxidative cardiac tissue damage upon reoxygenation. The lower mitochondrial content and respiration rate in ecto- vs endothermic tissues^41–43^ and the slightly reduced potential for ROS production with anoxia acclimation^13^ would also contribute to a lower mitochondrial ROS production upon reoxygenation in turtles. Whether this is reflected in lower ROS production *in vivo* remains to be tested. The capacity for H_2_O_2_ production is similar in turtle and mouse mitochondria (Fig. 6C) despite the lower respiration rate in turtle mitochondria (Fig. 6F) and so the high antioxidant capacity^44,45^ alone cannot explain the absence of anoxia/reoxygenation damage in turtles^13,14,45^. Our data so far suggests that ROS production upon reoxygenation is low due to decreased succinate accumulation and to the presence of ADP and that this may then largely explain the absence of oxidative damage.

In conclusion, the results of this study show that turtles are able to maintain heart function and survive very long periods of anoxia as well as reoxygenation due to a rewiring of heart metabolism. Our data indicates that the ability of turtles to balance ATP synthesis with consumption is a key factor. This maintains a functional adenine nucleotide pool, which keeps the heart functional during anoxia and blunts the potential for ROS production during reoxygenation. While succinate accumulates in turtles, it is to a much lower extent than in the ischemic mouse heart. Interestingly, in another model of extreme anoxia tolerance, the crucian carp, the ATP/ADP ratio also decreases to a new steady state level during anoxia, but in contrast to the turtle heart, neither alanine nor succinate accumulates^25^. This suggests that the unique ability of the crucian carp to convert pyruvate to ethanol^46^ effectively prevents pyruvate from entering the CAC, limiting the production of succinate and the potential for ROS production upon reoxygenation in this species. This supports our hypothesis that absent or limited succinate accumulation and preservation of ADP levels are central to protect the heart function of anoxia tolerant animals from oxidative damage upon reoxygenation.

## Materials and Methods

### Animals

Ten adult male and female *Trachemys scripta* (662 ± 19 g, mean ± SEM) from Nasco (Fort Atkinson, WI, USA) were kept in the animal facility at the Department of Bioscience, Aarhus University, for several months before they were fasted for 6 weeks and gradually acclimated to 5 °C over 6 weeks and then exposed to anoxia (N = 5) or normoxia (N = 5) for 9 days, as described in details elsewhere^13,47^. In addition, warm normoxic turtles (N = 3) were fed ad libitum and kept at 25 °C with free access to air. An overview of the animal treatment is given in Fig. 1A. Anoxic turtles were kept submerged in water continuously bubbled with N_2_, while normoxic turtles were allowed free access to air. Anoxic turtles were removed from the tanks with their heads pressed into their shields to limit their access to breathing. Turtles were euthanized with an overdose of 1 mg/kg pentobarbital injected in the supravertebral sinus. They were then kept at room temperature until the corneal reflex was gone after ~5 min and beheaded. The heart and blood from anoxic turtles had the characteristic dark colour after opening the plastron, indicating that tissues were only minimally reoxygenated when turtles were euthanized. Hearts were quickly dissected out and a ventricular sample (~50 mg) was immediately frozen in liquid N_2_ and stored at −80 °C before metabolomic analysis. All data was collected on heart ventricles which is referred to as turtle hearts.

Turtle heart metabolomics was compared to mouse heart metabolomics comparing control hearts (N = 6) to hearts exposed to ischemia for 30 min at 37 °C (N = 5). C57BL/6 female mice were purchased from Charles River Laboratories and maintained in specific-pathogen-free animal facilities with *ad libitum* access to food and water. Mice were anaesthetised with isoflurane (Abbott Laboratories, US) and O_2_ at 2 L/min. Heparin (100 µL bolus (25 iU) Leo Pharma A/S, Ballerup, Denmark) was administered intravenously into the inferior vena cava 1 min prior to prior to exsanguination by division of the inferior vena cava and aorta. Hearts were excised by division of the major vessels and rapidly frozen using Wollenberg clamps at liquid nitrogen (LN_2_) temperature. Control, normoxic mouse hearts were rapidly frozen taking ≤ 5 s to go from the beating heart to frozen. For ischemia, the excised heart was left in the abdomen of the animal, with the abdominal wound closed and the animal maintained at 37 °C using a relayed variable heat mat (Kent Scientific) for 30 min. At the end of the period of ischemia, hearts were clamp frozen using Wollenberg clamps as described above. Tissues were stored at −80 °C until analysis.

### Ethical statement

All turtle procedures were conducted according to the guidelines of the Danish Law on Animal Experiments and were approved by the Danish Animal Experiments Inspectorate under the permit 2015-15-0201-00544. All mouse procedures were conducted according to the guidelines of the Animals (Scientific Procedures) Act 1986 and were approved by the UK Home Office under the permit PPL 80/2638.

### Metabolite extraction and metabolomics analysis by LC-MS

Hearts were dissected as described^13^. Samples were homogenized in a grinder kept on dry ice and cooled with liquid nitrogen to keep the tissue frozen. Metabolites were then extracted from 20 mg homogenate in 500 µL extraction solution (50% methanol, 30% acetonitrile and 20% water). The extract was vortexed for 2 min and immediately centrifuged 16000 g at 4 °C for 10 min. The supernatant was then stored at −80 °C before LC-MS. LC-MS was conducted as previously described^9^. Turtle and mouse samples were analysed using the same LC-MS conditions. To allow for comparisons between groups, we used the web-based R-script https://metabolomics.cc.hawaii.edu/software/MetImp/ to pre-process the data. This imputes missing values using quantile regression imputation of left-censored data (QRLIC), which has been shown to be the best method for this type of missing values^48^. This corrected dataset was then used for all further analyses. The full dataset with and without this correction is given in Supplementary Table 1.

### Statistics

The metabolomics data was statistically analysed with the functions explore.data and univariate from the R package muma^49^. Statistical analysis of the data followed established parameters for determination of significance and data distribution for metabolomics data sets^49^. Each metabolite was tested for normality with the Shapiro-Wilks-test. If the p-value from the Shapiro-Wilks-test was greater than 0.05, the Welch’s t-test (normal distribution) or the Wilcoxon-Mann-Whitney test (non-normal distribution) was performed. p-values were corrected for multiple testing with the Benjamini-Hochberg correction and groups were considered significantly different when the final corrected p < 0.05. The full analysed datasets are given in Supplementary Table 2. All data is shown as means ± SEM.

### ATP/ADP assay

ATP and ADP concentrations were measured using a bioluminescence based Luciferase assay^50^. Frozen tissue samples were homogenised in ice-cold perchloric acid extracts (3% v/v HClO4, 2 mM Na_2_EDTA, 0.5% Triton X-100) and diluted to a concentration of 1 mg frozen tissue /ml. Samples and standards (400 µl) were pH neutralized using a potassium hydroxide solution (2 M KOH, 2 mM Na_2_EDTA, 50 mM MOPS). For ADP measurements, 250 µl neutralised sample was mixed with 250 µl ATP sulfurylase assay buffer to degrade remaining ATP (20 mM Na_2_MoO_4_, 5 mM GMP, 0.2 U ATP sulfurylase (New England Biolabs), in Tris-HCl buffer (100 mM Tris-HCl, 10 mM MgCl_2_ (pH 8.0)), incubated for 30 min at 30 °C and heat-inactivated at 100 °C for 5 min. Standards (100 µl), samples for ATP measurement (100 µl) or samples for ADP measurement (200 µl) (in duplicate) were added to 400 µl Tris-acetate (TA) buffer (100 mM Tris, 2 mM Na_2_EDTA, 50 mM MgCl2, pH 7.75) in luminometer tubes. 10 µl pyruvate kinase solution (100 mM PEP, 6 U pyruvate kinase suspension (Sigma # P1506)) were added to one set of samples for ADP measurement and incubated for 30 min at 25 °C in the dark to convert ADP to ATP. The other duplicate tube served as ADP ‘blank’ to each sample. ATP- dependent bioluminescence was measured using an AutoLumat LB-953-Plus multi-tube luminometer (Berthold) by addition of 100 µl Luciferase/Luciferin Solution (7.5 mM DTT, 0.4 mg/ml BSA, 1.92 µg luciferase/ml (SIGMA #L9506), 120 µM D-luciferin (SIGMA # L9504), made in TA buffer (25% v/v glycerol)), delivered via auto injection, protected from light. Luminescence was measured for 1 min post injection and the data quantified against standard curves.

### Mitochondrial isolation

Mitochondria were isolated from heart ventricles of warm normoxic turtles and control mice as described in detail elsewhere^13^. Briefly, the ventricles were washed in isolation buffer (250 mM sucrose, 10 mM HEPES, 1 mM EGTA, 0.5% bovine serum albumin (BSA), pH 7.4) and connective tissue was dissected free. The tissue was then carefully homogenised first with tweezers and scalpels and then with a Teflon pestle on ice. The homogenate was centrifuged at 700 g at 4 °C for 10 min to pellet cellular debris and the supernatant was filtered through two layers of cheese cloth into a new centrifuge tube and centrifuged twice at 9000 g at 4 °C for 10 min. The pellet was carefully rinsed with buffer to remove damaged mitochondria before resuspension in 200 µl isolation buffer without BSA. Protein content was determined with the Pierce 660 nm Protein Assay using BSA as a standard.

### Mitochondrial respiration and ROS production

Mitochondrial respiration rate and H_2_O_2_ production were measured on the Oroboros O2K oxygraph fitted with a Fluo2k unit. Isolated mitochondria (125 µg protein) were added to the respiration chambers containing 2 mL assay buffer (20 mM HEPES, 0.5 mM EGTA, 110 mM sucrose, 1.38 mM MgCl_2_, 20 mM taurine, 60 mM K-MES, 10 mM KH_2_PO_4_, pH 7.4) with 10 µM Amplex UltraRed, 1 U/mL HRP and 20 U/mL SOD calibrated to air. The FluO2k module was calibrated with injections of 0.1 µM H_2_O_2_ in the presence of mitochondria to correct for endogenous antioxidants. Following calibration, 10 mM succinate was added to induce RET, before 0.05–1.0 mM ADP was added to document the effect of ADP on ROS production. Maximal H_2_O_2_ production was quantified at [O_2_] ~250 µM with succinate. Differences were assessed by student’s t-test, and was considered significant when p < 0.05. Data is shown as means ± SEM.

## Supplementary information

Supplementary Table 1

Supplementary Table 2

## Data Availability

All data generated or analysed during this study are included in this published article and its Supplementary Information files.

## References

[1] Ultsch GR (1989). Ecology and physiology of hibernation and overwintering among freshwater fishes, turtles, and snakes. Biol. Rev..

[2] Ultsch GR (2006). The ecology of overwintering among turtles: where turtles overwinter and its consequences. Biol. Rev. Camb. Philos. Soc..

[3] Warren DE, Reese SA, Jackson DC (2006). Tissue glycogen and extracellular buffering limit the survival of red-eared slider turtles during anoxic submergence at 3 degrees C. Physiol. Biochem. Zool..

[4] Ultsch GR (1985). The viability of nearctic freshwater turtles submerged in anoxia and normoxia at 3 and 10 °C. Comp. Biochem. Physiol. Part A Physiol..

[5] Bickler PE, Buck LT (2007). Hypoxia tolerance in reptiles, amphibians, and fishes: Life with variable oxygen availability. Annu. Rev. Physiol..

[6] Jackson DC (2000). Living without oxygen: Lessons from the freshwater turtle. Comp. Biochem. Physiol. Part A.

[7] Ultsch GR, Jackson DC (1982). Long-term submergence at 3 °C of the turtle, *Chrysemys picta bellii*, in normoxic and severely hypoxic water. J. Exp. Biol..

[8] Jackson DC, Heisler N (1982). Plasma ion balance of submerged anoxic turtles at 3 °C: the role of calcium lactate formation. Respir. Physiol..

[9] Chouchani ET (2014). Ischaemic accumulation of succinate controls reperfusion injury through mitochondrial ROS. Nature.

[10] Murphy MP (2009). How mitochondria produce reactive oxygen species. Biochem. J..

[11] Chouchani ET (2016). A unifying mechanism for mitochondrial superoxide production during ischemia-reperfusion injury. Cell Metab..

[12] Buck LT (2000). Succinate and alanine as anaerobic end-products in the diving turtle (Chrysemys picta bellii). Comp. Biochem. Physiol. - B Biochem. Mol. Biol..

[13] Bundgaard, A., James, A. M., Joyce, W., Murphy, M. P. & Fago, A. Suppression of reactive oxygen species generation in heart mitochondria from anoxic turtles: the role of complex I S-nitrosation. *J. Exp. Biol*. jeb. 174391, 10.1242/jeb.174391 (2018). 10.1242/jeb.174391 PMC5963835 29496783

[14] Wasser JS, Meinertz EA, Chang SY, Lawler RG, Jackson DC (1992). Metabolic and cardiodynamic responses of isolated turtle hearts to ischemia and reperfusion. Am. J. Physiol..

[15] Rapatz GL, Musacchia XJ (1957). Metabolism of *Chrysemys picta* During Fasting and During Cold Torpor. Am. J. Physiol. Content.

[16] McCue MD (2007). Snakes survive starvation by employing supply- and demand-side economic strategies. Zoology.

[17] Wang T, Hung CCY, Randall DJ (2006). The comparative physiology of food deprivation: From Feast to Famine. Annu. Rev. Physiol..

[18] Almeida-Val VM, Buck LT, Hochachka PW (1994). Substrate and acute temperature effects on turtle heart and liver mitochondria. Am. J. Physiol..

[19] Taegtmeyer H, Hems R, Krebs HA (1980). Utilization of energy-providing substrates in the isolated working rat heart. Biochem. J..

[20] Drake AJ, Haines JR, Noble MIM (1980). Preferential uptake of lactate by the normal myocardium in dogs. Cardiovasc. Res..

[21] Jackson DC (2004). Surviving extreme lactic acidosis: The role of calcium lactate formation in the anoxic turtle. Respir. Physiol. Neurobiol..

[22] Kelly, D. A. & Storey, K. B. Organ-specific control of glycolysis in anoxic turtles. *Am. J. Physiol. - Regul. Integr. Comp. Physiol*. **255** (1988). 10.1152/ajpregu.1988.255.5.R774 2973250

[23] Galli GLJ, Lau GY, Richards JG (2013). Beating oxygen: chronic anoxia exposure reduces mitochondrial F_1_F_O_-ATPase activity in turtle (*Trachemys scripta*) heart. J. Exp. Biol..

[24] Stecyk JaW (2009). Correlation of cardiac performance with cellular energetic components in the oxygen-deprived turtle heart. Am. J. Physiol. Regul. Integr. Comp. Physiol..

[25] Lardon I (2013). 1H-NMR study of the metabolome of an exceptionally anoxia tolerant vertebrate, the crucian carp (*Carassius carassius*). Metabolomics.

[26] Nilsson GE, Lutz PL (2004). Anoxia Tolerant Brains. J. Cereb. Blood Flow Metab..

[27] Hochachka PW (1986). Defense Strategies against Hypoxia and Hypothermia. Science (80-.)..

[28] Hochachka PW, Buck LT, Doll CJ, Land SC (1996). Unifying theory of hypoxia tolerance: Molecular/metabolic defense and rescue mechanisms for surviving oxygen lack. Proc. Natl. Acad. Sci. USA.

[29] Bishop T, St-Pierre J, Brand MD (2002). Primary causes of decreased mitochondrial oxygen consumption during metabolic depression in snail cells. Am. J. Physiol. Regul. Integr. Comp. Physiol..

[30] Boutilier RG, St-Pierre J (2000). Surviving hypoxia without really dying. Comp. Biochem. Physiol. - A Mol. Integr. Physiol..

[31] Lutz, P. L. & Nilsson, G. E. Contrasting strategies for anoxic brain survival - glycolysis up or down. *J. Exp. Biol*. **200** (1997). 10.1242/jeb.200.2.411 9050250

[32] Brooks SP, Storey KB (1989). Regulation of glycolytic enzymes during anoxia in the turtle *Pseudemys scripta*. Am. J. Physiol..

[33] Rovetto MJ, Lamberton WF, Neely JR (1975). Mechanisms of glycolytic inhibition in ischemic rat hearts. Circ. Res..

[34] Depre C, Rider MH, Hue L (1998). Mechanisms of control of heart glycolysis. Eur. J. Biochem..

[35] Mochizuki S, Neely JR (1979). Control of glyceraldehyde-3-phosphate dehydrogenase in cardiac muscle. J. Mol. Cell. Cardiol..

[36] Hochachka PW, Owen TG, Allen JF, Whittow GC (1975). Multiple end products of anaerobiosis in diving vertebrates. Comp. Biochem. Physiol. - Part B Biochem..

[37] Hochachka PW, Storey KB (1975). Metabolic Consequences of Diving in Animals and Man. Science (80-.)..

[38] Peuhkurinen KJ, Takala TE, Nuutinen EM, Hassinen IE (1983). Tricarboxylic acid cycle metabolites during ischemia in isolated perfused rat heart. Am. J. Physiol..

[39] Kim J, Tchernyshyov I, Semenza GL, Dang CV (2006). HIF-1-mediated expression of pyruvate dehydrogenase kinase: A metabolic switch required for cellular adaptation to hypoxia. Cell Metab..

[40] Stecyk JAW, Overgaard J, Farrell AP, Wang T (2004). Alpha-adrenergic regulation of systemic peripheral resistance and blood flow distribution in the turtle *Trachemys scripta* during anoxic submergence at 5 degrees C and 21 degrees C. J. Exp. Biol..

[41] Hulbert, A. J. & Else, P. L. Evolution of mammalian endothermic metabolism: mitochondrial activity and cell composition. *Am. J. Physiol. - Regul. Integr. Comp. Physiol*. **256** (1989). 10.1152/ajpregu.1989.256.1.R63 2536249

[42] Else PL, Hulbert AJ (1985). An allometric comparison of the mitochondria of mammalian and reptilian tissues: The implications for the evolution of endothermy. J. Comp. Physiol. B.

[43] Else, P. L. & Hulbert, A. J. Comparison of the ‘mammal machine’ and the ‘reptile machine’: energy production. *Am. J. Physiol. - Regul. Integr. Comp. Physiol*. **240** (1981). 10.1152/ajpregu.1981.240.1.R3 6257122

[44] Willmore WG, Storey KB (1997). Glutathione systems and anoxia tolerance in turtles. Am. J. Physiol..

[45] Willmore WG, Storey KB (1997). Antioxidant systems and anoxia tolerance in a freshwater turtle *Trachemys scripta elegans*. Mol. Cell. Biochem..

[46] Fagernes CE (2017). Extreme anoxia tolerance in crucian carp and goldfish through neofunctionalization of duplicated genes creating a new ethanol-producing pyruvate decarboxylase pathway. Sci. Rep..

[47] Jensen FB, Hansen MN, Montesanti G, Wang T (2014). Nitric oxide metabolites during anoxia and reoxygenation in the anoxia-tolerant vertebrate *Trachemys scripta*. J. Exp. Biol..

[48] Wei R (2018). Missing Value Imputation Approach for Mass Spectrometry-based Metabolomics Data. Sci. Rep..

[49] Gaude E (2013). *muma*, An R Package for Metabolomics Univariate and Multivariate Statistical Analysis. Curr Metabolomics.

[50] Strehler, B. L. Adenosine-5′-triphosphate and Creatine Phosphate Determination with Luciferase. *Methods Enzym. Anal*. 2112–2126 (1974).

